# Safety and efficacy of intermittent fasting with or without exercise in people living with overweight or obesity and type 2 diabetes—The INTERFAST‐3 study design

**DOI:** 10.1111/dme.70328

**Published:** 2026-04-15

**Authors:** Caren Sourij, Alexander Müller, Abdullah Al‐Baghdadi, Faisal Aziz, Norbert J. Tripolt, Peter N. Pferschy, Eva Andritz, Harald Kojzar, Vanessa Stadlbauer, Sabrina Leal Garcia, Harald Sourij

**Affiliations:** ^1^ Division of Cardiology Medical University of Graz Graz Austria; ^2^ Cardiometabolic Trials Unit, Division of Endocrinology and Diabetology Medical University of Graz Graz Austria; ^3^ Division of Gastroenterology and Hepatology Medical University of Graz Graz Austria; ^4^ Department for Medical Psychology, Psychosomatic and Psychotherapy Medical University of Graz Graz Austria

**Keywords:** CGM (continuous glucose monitoring), exercise, intermittent energy restriction, intermittent fasting, lifestyle, obesity, type 2 diabetes mellitus

## Abstract

**Aims:**

Lifestyle modification, including caloric restriction and exercise, is fundamental in the treatment of people living with overweight and type 2 diabetes mellitus (T2D). The overall aim of lifestyle intervention schemes is to reduce body weight and improve glycaemic control to reduce the future risk of diabetes‐associated complications. The aim of this study is to determine whether intermittent energy restriction, exercise or their combination is best suited for reaching these targets in individuals with T2D not treated with insulin.

**Methods:**

This randomized, controlled, monocentric parallel‐group trial is designed to investigate participants living with T2D, body mass index >27 kg/m^2^, HbA1c ≥53 mmol/mol (≥7.0%) and ≤86 mmol/mol (≤10.0%) and without insulin therapy. Participants are equally and randomly allocated to one of four study groups: (1) intermittent energy restriction group, (2) exercise group, (3) combined intermittent energy restriction and exercise group and (4) control group. The intervention phase lasts 12 weeks, followed by a two‐year follow‐up phase. In addition to assessing body weight and glycaemic parameters, resting energy expenditure (REE) and body composition are measured. An oral glucose tolerance test is carried out at baseline and at the end of the intervention. Additionally, stool samples for microbiome analyses and individual‐related outcomes are collected, and all participants are equipped with continuous glucose monitoring (CGM). The primary outcome measure is the change in bodyweight from baseline to day 84.

**Trial Registration:**

The study was registered at DRKS (Deutsches Register Klinischer Studien—German Clinical Trial Register DRKS‐ID: DRKS00032036)—Date of registration: 11.10.2023.

**What's new?**

- Lifestyle intervention is a cornerstone of therapy for type 2 diabetes mellitus (T2D), aiming to improve glucose metabolism and reduce body weight.
- Intermittent energy restriction was proven to be a safe and effective method to improve glycaemic control and body weight.
- Comparative effectiveness trials of intermittent energy restriction and regular physical activity or a combination of both in people with T2D are scarce.
- The INTERFAST‐3 study aimed to determine whether intermittent energy restriction, exercise or their combination is best suited for reducing bodyweight and improving glucose metabolism in individuals with T2D.

## 1 INTRODUCTION

Type 2 diabetes mellitus (T2D) is a chronic metabolic disorder characterized by persistent hyperglycaemia due to a progressive loss of β‐cell insulin secretion commonly on the background of insulin resistance.^1^, ^2^ The International Diabetes Federation (IDF)^3^ estimates the prevalence of diabetes in 2024 at approximately 590 million adults, which provides to be a global burden on public health and projections suggest that the number of people with diabetes could rise beyond 850 million until 2050.^3^ T2D causes undesirable health consequences in multiple areas of the human body such as increasing the incidence of serious disorders affecting eyes, kidneys, somatic nerves, autonomic nerves, feet, heart,^4^, ^5^ brain, blood vessels, liver, skeleton and other tissues.^3^, ^6^, ^7^, ^8^

Overweight and obesity represent a multifactorial disease in which, besides genetic predisposition, exposure to an environment characterized by sedentary behaviour and high‐calorie intake contributes as a significant risk factor for the development of T2D.^9^, ^10^ The excessive free fatty acid release by adipose tissue leads to a decrease in insulin sensitivity of muscles, adipose tissue and liver, followed by increased glucose levels, insulin resistance and T2D.^11^, ^12^, ^13^, ^14^

As a result, institutions such as the ADA (American Diabetes Association) or the WHO (World Health Organization) recommend body weight reduction through calorie restriction, along with at least 150 min of moderate‐intensity aerobic physical activity or 75 min of vigorous‐intensity aerobic physical activity spread over at least 3 days per week.^15^, ^16^

Dietary modifications aiming to reduce body weight represent one cornerstone of treatment of people living with obesity and type 2 diabetes for blood glucose, weight and cardiovascular risk‐factor management.^17^ Intermittent energy restriction (IER), defined as periods of caloric restriction alternating with periods of ad libitum eating has been proposed as an umbrella term including intermittent fasting and time‐restricted eating.^18^ While previous data has demonstrated that IER regimens have similar effects on anthropometric and cardiometabolic risk factors^19^ current diabetes guidelines highlight the simplicity of IER for people looking for a practical dietary weight loss tool, who are challenged by continuous caloric restriction regimens.^20^

Although previous studies have demonstrated beneficial effects of dietary restrictions and exercise individually in people with T2D, data on the combined impact of an IER regimen and physical activity compared with each lifestyle intervention alone remains scarce.^21^ Understanding the interaction between exercise and intermittent energy restriction is therefore crucial in order to find the best lifestyle intervention strategies. Hence, the aim of this ongoing study is to assess the safety and efficacy of IER, structured exercise and their combination in people living with overweight/obesity and T2D.

## 2 METHODS

### 2.1 Study hypothesis

**FIGURE 1:**
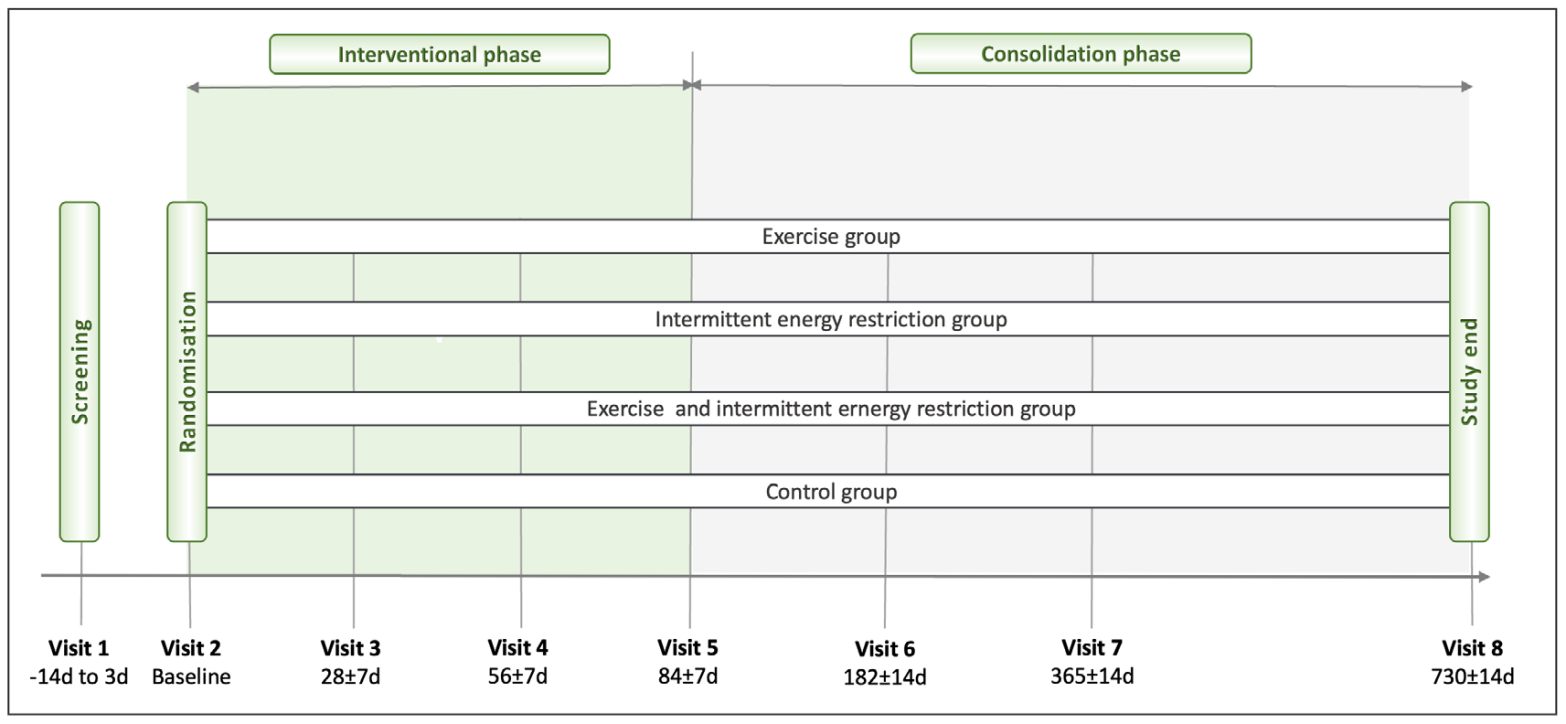
Study overview.

Caloric restriction and exercise are keystones of anti‐hyperglycaemic therapy. We hypothesize that the combination of both interventions is safe and most effective for bodyweight reduction and HbA1c lowering in people living with overweight and T2D (Figure 1).

### 2.2 Study design

This randomized, controlled, parallel‐arm trial is designed to compare the effects of intermittent energy restriction, structured exercise and their combination in people living with overweight/obesity and T2D. The study visits take place at the Cardiometabolic Trials Unit at the Medical University of Graz (Table 1).

**TABLE 1:** Visit schedule.

	Visit 1 (−14 to 3 days)	Visit 2 Baseline	Visit 3 (28 ± 7 days)	Visit 4 (56 ± 7 days)	Visit 5 (84 ± 7 days)	Visit 6 (184 ± 14 days)	Visit 7 (365 ± 14 days)	Visit 8 (730 ± 21 days)
Informed consent	X							
Inclusion/exclusion criteria	X							
Randomization		X						
Demography, medical history	X							
Concomitant medication	X	X	X	X	X	X	X	X
Vital signs	X	X	X	X	X	X	X	X
Height	X							
Weight	X	X	X	X	X	X	X	X
Physical examination	X				X			
Electrocardiography (ECG)	X							
Resting energy expenditure (REE)		X			X			
Blood sampling		X	X	X	X	X	X	X
Biobanking		X	X	X	X	X	X	X
Oral glucose tolerance test		X			X			
Adverse events			X	X	X	X	X	X
Bioelectrical impedance analysis		X	X	X	X	X	X	X
6‐min walk test (6‐MWT)		X			X			
Food frequency questionnaire (FFQ)		X	X	X	X	X		
International physical activity questionnaire (IPAQ)		X	X	X	X	X		
Start of CGM use		X						
End of CGM use					X			
Lifestyle counselling		X			X			
Eating disorder examination (EDE‐Q)		X	X	X	X	X		
Sleep quality questionnaire		X	X	X	X	X		
Food craving inventory		X	X	X	X			
Beck depression questionnaire (BDI)		X	X	X	X			
Application/removal movisens		X	X	X	X			
Faeces sampling		X			X			
Whole blood aggregation		X	X	X	X			
Heart rate monitoring^*^		X						
Removal heart rate monitoring					X			
BODPOD‐test		X			X			
PMBC blood draw		X			X	X		
Measurements of handgrip strength		X			X			
MoCA (montreal cognitive assessment)		X			X			
Symbol digit modalities test (SDMT)		X			X			

^*^ In people randomized to the exercise or combined intervention group.

The study was conducted in accordance with good clinical practice guidelines (Declaration of Helsinki, ICH‐GCP), and ethics approval was obtained from the ethics committee of the Medical University of Graz (EK 35‐337 ex 22/23). All participants provide written informed consent prior to participating in the study.

#### 2.2.1 Study population

All study participants are aged 18 to 75 years old with established T2D with HbA1c levels ≥7.0% (≥53 mmol/mol) and ≤10.0% (≤86 mmol/mol). They have a BMI >27.0 kg/m^2^ and maintained a stable body weight for the preceding 3 months.

Individuals who are willing and able to comply with study procedures, including the prescribed fasting regime and exercise protocol, are included in the study.

The key exclusion criteria contain any other form of diabetes mellitus than T2D, history of diabetic ketoacidosis, treatment with insulin or sulfonylurea, known active malignancy, pregnancy or any chronic medical condition that could interfere with the interpretation of study outcomes. Additional exclusions include conditions that prevent participation in the exercise programme, acute coronary syndrome or stroke within the last 6 months.

#### 2.2.2 Recruitment

People living with overweight and T2D without insulin therapy are recruited from the Diabetes Registry for Biomarker Research Graz, via advertisement in local newspapers, social media platforms like Facebook and pamphlets distributed to general practitioners' surgeries.

#### 2.2.3 Randomization

Participants are randomized in a 1:1:1:1 ratio to one of the four intervention groups: Intermittent energy restriction alone, structured exercise alone, combination of intermittent energy restriction and exercise or no‐intervention control group. Randomization is performed using the Randomizer Software (http://www.randomizer.at), according to schedule generated by an independent statistician.

#### 2.2.4 Intervention

The active study intervention period is 84 ± 7 days followed by a consolidation period for 21 months to further investigate sustainability of the interventions. All arms are treated according to the Austrian diabetes guidelines,^22^ which are in line with the EASD/ADA consensus statement.^23^

- Intermittent energy restriction

Participants of the Intermittent energy restriction group are instructed to consume only 25% of the recommended daily caloric intake (caloric intake is fixed at a maximum of 600 kcal) within a time window of 6 h on fasting days (Monday, Wednesday, Friday) and eat food as desired on alternating feast days (Tuesday, Thursday, Saturday, Sunday). As such, on fast days, participants are fasting for approximately 18 h (6 h eating window on fasting days). Participants of this group receive dietary counselling at baseline.

b Exercise group

Participants assigned to the exercise group participate in a moderate‐intensity physical activity program three times a week for 12 weeks. This program comprises an indoor cycling unit, a nordic walking session and a structured body weight aerobic session, all performed at moderate intensity and supervised by a qualified exercise physiologist. All training sessions are monitored by a wrist‐worn fitness tracker (movisens GmbH, Germany). All exercise sessions last 60 min including a 10‐min warm‐up performed at 40–60% of maximum heart rate (HR_max_), followed by a cool‐down phase. Heart rate (HR) during exercise sessions will be recorded using a HR monitor (Polar, Finland).

c Combined exercise and Intermittent energy restriction

Participants in the combined intervention group follow both the IER regime and the exercise program, with exercise sessions scheduled on the IER days.

d Control group

Participants in the control group are instructed to maintain their usual lifestyle habits and not alter their eating or physical activity habits throughout the 12‐week intervention period. No specific lifestyle counselling session is provided to this group.

Participants of all four groups are instructed on how to apply and use the CGM device and how glucose readings and trend arrows are to be interpreted.

### 2.3 Sample size estimation

The sample size estimation is based on the analysis of covariance (ANCOVA) approach and data from our INTERFAST‐2 study that investigated exercise and fasting interventions in people with T2D treated with insulin.^24^ Based on INTERFAST‐2 data, we assumed the mean ± standard deviation body weight of 104 ± 13 kg at baseline, correlation (r) of 0.95 from baseline to follow‐up and the mean difference in weight loss of 5.0 ± 4.1 kg from baseline to follow up between intervention arms. Considering a 2‐sided type 2 error rate of 5% and a power of 90%, a total of at least 60 completers is required (15 in each study arm). The study discontinuation rate is expected to be 15%; therefore, the overall sample size is estimated to be 70 participants but could be higher to satisfy the requirement of 15 completers in each study arm.

### 2.4 Outcomes

The primary outcome of this study is the change in body weight from baseline to end of the intervention phase (i.e., 12 weeks). Key secondary outcomes comprise change in Time In Range (TIR) [70─180 mg/dL; 3.9─10.0 mmol/L] from baseline to end of intervention and change in the composite of improvement in TIR of at least 5%, weight reduction of at least 3% and stable or reduced glucose‐lowering treatment at end of intervention. Safety endpoints include difference in the number of severe adverse events and differences in TBR1 [54─<70 mg/dL; 3.0─<3.9 mmol/L] and TBR2 [<54 mg/dL; <3.0 mmol/L].

The full list of exploratory secondary outcomes can be found in Table S1.

### 2.5 Investigations

#### 2.5.1 Medical history

A medical history is recorded at screening visit to capture illnesses, disorders and medications. This information is updated on all follow‐up visits. Body weight, height and blood pressure are measured at all visits.

#### 2.5.2 Laboratory assessments

Blood samples are obtained at all visits after a minimum of 8 h overnight fasting and processed by the local laboratory using standard methods for routine tests. Participants may take their regular morning medication but are asked not to take any of their diabetes medications in the morning of their study visit.

Furthermore, blood samples are collected for biobanking at most visits and stored at the Biobank of the Medical University of Graz at −80°C.

#### 2.5.3 Bioelectrical impedance analysis (BIA)

BIA (BIACORPUS^RX4000^, MEDICAL HealthCare GmbH) is used to determine the distribution of the individual body components (fluids, muscle mass and fatty substance) in the study participants.

#### 2.5.4 Nutrition counselling

Nutrition counselling is performed by a professional dietician on site and recommendations and examples for menus on the fasting days are given to the participants.

#### 2.5.5 Indirect calorimetry

The resting energy expenditure (REE) is measured by indirect calorimetry (IC) (Cortex, Germany) before and after the study intervention. Oxygen consumption and carbon dioxide production is measured, and energy expenditure is calculated by the Weir formula.^25^

#### 2.5.6 Fitness tracking

To assess total energy expenditure, a fitness tracker (movisens GmbH, Germany) is worn continuously on the non‐dominant wrist throughout the intervention period.

#### 2.5.7 Questionnaires

All participants of this study are asked to fill out 6 different Questionnaires: (1) the Food Frequency Questionnaire (FFQ), where frequency and portion size of consumption of food items are asked according to specified categories, (2) the International Physical Activity Questionnaire (IPAQ). This tool was developed for measuring physical activity and is used in the current study to record the time spent being physically active in the last 7 days, (3) The Eating Disorder Examination Questionnaire (EDE‐Q) is a 28‐item self report questionnaire, adapted from the semi‐structured interview, the Eating Disorder Examination (EDE). The questionnaire is designed to assess the range, frequency and severity of behaviours associated with a diagnosis of an eating disorder, (4) The Pittsburgh Sleep Quality Index (PSQI) is a self report questionnaire that assesses sleep quality over a 1‐month time interval, (5) Food Craving Inventory is a reliable and valid self‐report measure of general and specific food cravings, (6) The Beck Depression Inventory (BDI), a 21‐item self report rating inventory, is used to measure characteristic attitudes and symptoms of depression, and (7) A questionnaire is used to assess the satisfaction with the CGM system.

Cognitive function test available at cognitivefunctiontest.info provided by Food for Brain (UK) will also be applied.

#### 2.5.8 Continuous glucose monitoring (CGM)

CGM (Abbott FreeStyle Libre 2 device, Abbott Diabetes Care, Almeda, CA, USA) is used throughout the intervention phase.

#### 2.5.9 Six‐minute walking test (6‐MWT)

The 6‐MWT is performed on an indoor course, and participants are instructed to walk as quickly as possible for 6 min. The distance walked is measured to the closest metre.

#### 2.5.10 Oral glucose tolerance test (oGTT)

Participants attend the Cardiometabolic Trials Unit in the morning after an overnight fast. The oral glucose tolerance test (oGTT) is performed at the beginning and at the end of the 12‐week intervention phase. Participants consume 75 g of glucose diluted in 250 mL of water (Glucoral 75 citron, Germania Pharmazeutika, Vienna) within ≤5 min; blood is sampled every 30 min for 2 h. The oGTT is performed after an overnight fast following an eating day (for the IER group alone and the combination group of IER and exercise).

#### 2.5.11 Faeces sampling and microbiome analysis

Sampling is performed using stool collection tubes (Sarstedt, Nümbrecht, Germany) at start and end of intervention phase. Bacterial DNA will be extracted from stool samples using the MagNA Pure LC DNA Isolation Kit III (Roche). The 16S rRNA gene will be amplified in a PCR reaction, sequenced with next‐generation sequencing technology (Roche Genome Sequencer FLX or Illumina MiSeq) and analysed by an in‐house R pipeline.

#### 2.5.12 Measurements of handgrip strength

Handgrip strength is measured using the JAMAR Hydraulic Hand Dynamometer (Patterson Medical) as an indicator of overall muscle strength and physical function. It is widely recognized as a reliable proxy for total body strength and has been shown to correlate strongly with functional performance across multiple populations.^26^, ^27^

### 2.6 Investigations in subgroups

In a subgroup of participants, additional investigations are carried out. These investigations include the MoCA (Montreal Cognitive Assessment) screening, the Symbol Digit Modalities Test (SDMT), BODPOD as an additional method to measure body composition and the Doubly labelled water methods for measuring total energy expenditure. A complete list of the additional investigations can be found in the supplements (Table S2).

### 2.7 Glycaemic management

Participants in this randomized clinical trial may start additional non‐randomized medication according to the guidelines of the Austrian Diabetes Association^22^ if HbA1c remains >7.5% (>58 mmol/mol) at visit 3 or 4.

### 2.8 Statistics

A detailed statistical analysis plan will be finalized before locking the database. All statistical analyses will be conducted in R version 4.5.2. The primary analysis will be conducted on the ITT population and sensitivity analysis will be conducted in the PP population. In descriptive analysis, continuous variables will be summarized as mean ± SD and categorical variables as frequencies and percentages (%) for the overall trial population and by each trial arm, respectively. The primary outcome of mean change in weight over visits between trial arms will be analyzed using the mixed effects model (MMRM) with an unstructured covariance matrix for modelling within‐subject residuals and considering maximum likelihood estimation under the missing‐at‐random assumption. The MMRM will include intervention groups, visit and intervention‐by‐visit interaction as fixed effects. In addition, baseline body weight, baseline body weight–visit interaction, age, sex, duration of diabetes, use of GLP‐1 receptor agonist and glucose‐lowering medications will be added as adjustment covariates in the MMRM model.

The marginal mean for change in body weight with 95% CIs will be estimated from the MMRM model for each intervention group. Family‐wise error for multiple comparisons will be controlled by employing Dunnett's approach and the fixed sequence gate‐keeping method. In step 1, pairwise comparisons of each active intervention group with the control group will be conducted using a 2‐sided alpha level of 0.05 and Dunnett's adjusted confidence intervals. In step 2, if any intervention group is found to be significantly different from the control group (*p* < 0.05), exploratory pairwise comparisons between active intervention groups will be conducted, and multiplicity adjustment will be carried out using Holm's method. The primary results will also be reported as model‐derived percentage change difference from baseline between intervention groups.

Key secondary outcomes will be analysed using similar statistical modelling, fixed sequence and family‐wise error control strategies. Specifically, change in TIR will be analysed using the MMRM model. The percentage of participants achieving composite outcome of TIR increase (≥5%), body weight reduction (≥3%) and stable or reduced glucose‐lowering treatment over visits will be analysed using generalized estimating equations (GEE) with binomial distribution and identify link function and considering the exchangeable correlation structure. Robust standard errors will be estimated and results will be reported in terms of differences in marginal probabilities and marginal odds ratios along with 95% CIs.

The prespecified subgroup analyses will also be conducted to evaluate the heterogeneity of intervention effect on primary outcome with respect to covariates including duration of diabetes, baseline body weight, GLP‐1 agonist use and HbA1c. For continuous variables, both continuous values as well as relevant cut‐offs will be used. For each subgroup analysis, interaction term for the subgroup variable with intervention will be added in the primary MMRM model and both model estimates with p‐interactions and marginal means for each subgroup will be reported. The subgroup analyses will be exploratory and therefore not be corrected for multiplicity.

### 2.9 Adherence to protocol

Adherence to IER is calculated as the number of fasting days (more than 75% caloric reduction); for the exercise group, adherence is calculated as the number of exercise sessions performed divided by the total number of planned IER or exercise sessions, respectively. 80% or more is considered adherent to the study protocol.

### 2.10 Gender

The INTERFAST‐3 study aims to enrol an equal number of women and men participants.

### 2.11 Study status

As of November 4th, 2025, 65 individuals (93%) have been randomized.

### 2.12 Registration

The study was registered at DRKS (Deutsches Register Klinischer Studien—German Clinical Trial Register—DRKS‐ID: DRKS00032036; date of registration: 11.10.2023).

## 3 STRENGTHS AND LIMITATIONS /DISCUSSION

Weight reduction is one of the cornerstones of T2D therapy in people living with overweight or obesity. Previously we investigated the effects of intermittent energy restriction in people with insulin treated T2D,^24^ demonstrating that 3 non‐consecutive days of IER per week over 12 weeks improved HbA1c, reduced body weight and led to a total daily insulin dose reduction. Physical activity has also been shown to improve glycaemic control^28^ and is therefore recommended in T2D management guidelines all over the world.^15^, ^23^ However, data on potential beneficial effects of combining IER with structured exercise on weight loss and glycaemic control remain scarce.

The greatest strength of this study is its randomized controlled design together with CGM data collected throughout the active intervention phase for all groups, as well as the control group. Besides analysing the glycaemic excursions during fasting and/or exercise with CGM, participants have the possibility to learn how eating habits and exercise affect blood glucose levels. As all groups use CGMs, the effects observed in the IER, exercise and combination groups are controlled for this CGM inherent effect.

A challenge of this study is the evaluation of HbA1c reduction during the active phase, as additional non‐randomized medication including GLP‐1 receptor agonists may be started if HbA1c remains >58 mmol/mol (>7.5%) at visit 3. However, not intensifying the anti‐hyperglycaemic treatment during the active phase was considered unethical. We have defined the change in glucose‐lowering medication (decrease, no change, increase) as secondary outcome to specifically look into this topic and will collect detailed information on changes in metabolically active pharmacological agents. Another limitation is the duration of the active phase of only 12 weeks. However, we perform follow‐up visits up to 2 years after randomization to evaluate whether participants continued to implement the intervention in their daily routine and how sustainable a potential effect observed after the 12‐week active phase was.

One can argue that combining a time‐restricted eating and a caloric restriction component to the chosen dietary intervention will not allow one to separate out if a potential beneficial effect is due to one or the other. However, this is not the intention of the current study, which focuses on the changes in clinically relevant outcomes of a simple‐to‐follow dietary intervention rather than in‐depth mechanistic investigations.

The INTERFAST‐3 trial aims to investigate the interplay between IER and exercise in individuals with non‐insulin‐treated T2D, thereby helping to find the best tailored lifestyle intervention for each individual living with T2D.

## AUTHOR CONTRIBUTIONS

HS designed the study and received funding. FA performed statistical analysis planning. VS, PNP, NJT and SLG contributed to the design. CS, AM, HK and AAB performed the study visits. EA does dietary counselling and the evaluation of dietary records. CS wrote the first draft, and all authors reviewed and contributed to the final manuscript.

## FUNDING INFORMATION

Harald Sourij was supported by research funding from the Austrian Science Fund (Grant‐DOI 10.55776/KLP3413324, Grant‐DOI 10.55776/PIN8074224).

## CONFLICT OF INTEREST STATEMENT

All authors have no conflicts to declare.

## Supporting information


**Table S1.** Full list of exploratory secondary outcomes.
**Table S2.** List of subgroup investigations.
