# Supplementary material for: Safety and efficacy of intermittent fasting with or without exercise in people living with overweight or obesity and type 2 diabetes—The INTERFAST‐3 study design
Source: Diabet Med. 2026 Apr 15;43(7):e70328. doi: 10.1111/dme.70328 (PMC13257894; doi:10.1111/dme.70328)
Supplement: Supplementary file 1 — Table S1. Full list of exploratory secondary outcomes. Table S2. List of subgroup investigations. [file DME-43-e70328-s001.docx]

**Supplements**

**Supplemental table 1**

Full list of exploratory secondary outcomes

Abbr: TBR time below range, TAR time above range

| - Differences in TBR 2 [<54mg/dl; < 3.0 mmol/L] from baseline to day 84 (safety) |
| --- |
| - Differences in TBR1 [54─<70mg/dL; 3.0─<3.9mmol/L]) from baseline to day 84 (safety) |
| - Differences in TAR 1 (181─250 mg/dL; 10.1─13.9 mmol/L) from baseline to day 84 |
| - Differences in TAR 2 (>250 mg/dL; >13.9 mmol/L) from baseline to day 84 |
| - Differences in glycaemic variability (%CV, SD) from baseline to day 84 |
| - Differences in numbers of hypoglycaemia (<70 mg/dL; <3.9 mmol/L) with a duration >15 min as assessed via continuous glucose monitoring (CGM) from baseline to day 84 |
| - Differences in the change of glycated haemoglobin (HbA1c) from baseline to day 84 |
| - Differences in body composition (body mass, fat mass, lean mass) from baseline to day 28, 56 and day 84 |
| - Differences in resting metabolic rate (RMR) from baseline to day 84 |
| - Differences in the change of quality of life from baseline to day 84 |
| - Differences in the change of calorie intake from baseline to day 84 |
| - Differences in the change of physical activity (IPAQ, Movisens) from baseline to day 84 |
| - Differences in the change of fasting glucose from baseline to day 84 |
| - Differences in the incremental area under the curve (iAUC) for glucose from day 0 to day 84 during the oGTT |
| - Differences in the incremental area under the curve (iAUC) for insulin from day 0 to day 84 during the oGTT |
| - Differences in the trapezoidal area under the curve (tAUC) for glucose from day 0 to day 84 during the oGTT |
| - Differences in the trapezoidal area under the curve (tAUC) for insulin from baseline to day 84 during the oGTT |
| - Differences in the peak plasma glucose concentration during oGTT from baseline to day 84 |
| - Differences in the change of insulin resistance (HOMA-IR, insulinogenic index) from baseline to day 84 |
| - Differences in the change of insulin sensitivity (Matsuda-Index) from baseline to day 84 |
| - Differences in sleep quality and duration (PSQI Questionnaire) from baseline to day 84 |
| - Differences in the 6 MWT (6-minutes walking test) from baseline to day 84 |
| - Differences in the microbiome composition from baseline to day 84 |
| - Differences in HbA1c from Baseline to day 730 |
| - Change in the composite of improvement HbA1c of 0.5% (5mmol/mol), weight reduction of at least 3% and stable or reduced glucose lowering treatment at day 730 |
| - Change in antihyperglycaemic medication (decrease, no change, increase) from baseline to day 84 |
| - Change in antihyperglycaemic medication (decrease, no change, increase) from baseline to day 730 |
| - Change in Measurements of handgrip strength |
| - Change in MoCA (Montreal Cognitive Assessment) |
| - Change in Symbol Digit Modalities Test (SDMT) |
| - Difference in the number of severe adverse events (safety) |

**Supplemental table 2**

List of subgroup investigations

| 1. MoCA (Montreal Cognitive Assessment)   The MoCA is a cognitive screening tool designed to detect mild cognitive impairment (MCI), Alzheimer's disease, and other forms of early dementia. The test evaluates multiple cognitive domains, including short-term memory recall, visuospatial abilities (such as clock drawing and cube copying), executive functions (like trail making and verbal abstraction), attention, concentration, working memory, language and orientation to time and place. |
| --- |
| 1. Symbol Digit Modalities Test (SDMT)   The Symbol Digit Modalities Test (SDMT) is a neuropsychological tool designed to assess cognitive processing speed, sustained attention, visual scanning, and motor speed. The test is particularly sensitive to detecting subtle cognitive impairments and is frequently used in the evaluation of neurological conditions such as multiple sclerosis (MS), traumatic brain injury (TBI), stroke, dementia, and Parkinson's disease. |
| 1. BODPOD (Subgroup of 30 participants)   The BODPOD is a device used to measure body composition, particularly body fat percentage and muscle mass. It operates based on the principle of air displacement plethysmography, where the body's volume is determined by measuring the air pressure in a closed chamber. The BodPod is considered precise and non-invasive, and it is  commonly used in sports and health fields. It aims to demonstrate changes in body composition that may occur with weight loss during a physical activity or fasting intervention in the INTERFAST Study 3 |
| 1. Doubly labelled water (DLW, Subgroup of 20 participants)   In this investigation, two methods for measuring total energy expenditure (TEE) using the doubly labeled water technique will be compared. This method determines TEE by tracking the different elimination rates of the stable isotopes deuterium (^2^H) and oxygen-18 (^18^O). |
| 1. PBMC (Subgroup of 30 patients)   To test whether the P72R p53 polymorphism is linked to the degree of weight loss and weight regain in the INTERFAST 3 cohort, we will perform targeted sequencing from PBMCs at visits 2,5 and 6. |
